# Health Care Professionals’ Evidence-Based Medicine Internet Searches Closely Mimic the Known Seasonal Variation of Lyme Borreliosis: A Register-Based Study

**DOI:** 10.2196/publichealth.6764

**Published:** 2017-04-11

**Authors:** Samuli Pesälä, Mikko J Virtanen, Jussi Sane, Jukkapekka Jousimaa, Outi Lyytikäinen, Satu Murtopuro, Pekka Mustonen, Minna Kaila, Otto Helve

**Affiliations:** ^1^ University of Helsinki Helsinki Finland; ^2^ National Institute for Health and Welfare Helsinki Finland; ^3^ Duodecim Medical Publications Ltd Helsinki Finland; ^4^ Public Health Medicine Helsinki University Hospital University of Helsinki Helsinki Finland; ^5^ Children's Hospital Helsinki University Hospital University of Helsinki Helsinki Finland

**Keywords:** search engine, evidence-based medicine, information systems, public health surveillance, Lyme borreliosis

## Abstract

**Background:**

Both health care professionals and nonprofessionals seek medical information on the Internet. Using Web-based search engine searches to detect epidemic diseases has, however, been problematic. Physician’s databases (PD) is a chargeable evidence-based medicine (EBM) portal on the Internet for health care professionals and is available throughout the entire health care system in Finland. Lyme borreliosis (LB), a well-defined disease model, shows temporal and regional variation in Finland. Little data exist on health care professionals’ searches from Internet-based EBM databases in public health surveillance.

**Objective:**

The aim of this study was to assess whether health care professionals’ use of Internet EBM databases could describe seasonal increases of the disease and supplement routine public health surveillance.

**Methods:**

Two registers, PD and the register of primary health care diagnoses (Avohilmo), were used to compare health care professionals’ Internet searches on LB from EBM databases and national register-based LB diagnoses in order to evaluate annual and regional variations of LB in the whole country and in three selected high-incidence LB regions in Finland during 2011-2015.

**Results:**

Both registers, PD and Avohilmo, show visually similar patterns in annual and regional variation of LB in Finland and in the three high-incidence LB regions during 2011-2015.

**Conclusions:**

Health care professionals’ Internet searches from EBM databases coincide with national register diagnoses of LB. PD searches showed a clear seasonal variation. In addition, notable regional differences were present in both registers. However, physicians’ Internet medical searches should be considered as a supplementary source of information for disease surveillance.

## Introduction

Traditionally, many syndromic surveillance systems have collected epidemiological data from health care professionals’ clinical encounters when predicting several disease epidemics, such as influenza [1]. This worldwide severe disease showing seasonal and geographical variation can be predicted by using Internet search trends [1-3]. Internet-based surveillance systems have allowed good congruence with traditional (data submitted by public health authorities) surveillance approaches for monitoring emerging infectious diseases of public health concern [1,3]. To improve early detection of influenza, Google search queries were used to track influenza-like disease in population [1,2]. Because certain searches from Google correlated highly with medical visits related to influenza-like symptoms, influenza activity could be estimated geographically [2]. However, when reassessed, the reliability of the surveillance tool was shown to be problematic and to contain substantial flaws, especially in regard to assessing the correct timing and location [4]. Therefore, it has been commented that the use of near-real time electronic health data and computational methods should be incorporated [4]. The models such as Internet-based influenza monitoring have not, however, included the characterization of the populations performing the searches, also comprising nonprofessionals. Internet search engine queries and the data from social media can be combined to detect infectious diseases as well [5]. Generally, for framing the methods on health-related Internet information and epidemiological data, two terms are used: infodemiology and infoveillance. Infodemiology can be determined as a discipline inside public health informatics that studies information in an electronic medium or in a population aiming to inform public health and public policy [6]. When infodemiology data are used for surveillance purposes, the term is called infoveillance [6].

Health care professionals’ use of CD-ROM–based medical searches as a tool for early detection of epidemics has been studied earlier in Finland during 1995 using the National Infectious Diseases Register (NIDR) as a reference register [7]. Microbiological laboratories notify diagnostic findings electronically to NIDR. Medical professionals use electronic sources to provide appropriate answers to clinical questions, such as Internet sites and search engines [8]. For doctors and nurses, the main reasons to seek Internet-based evidence are patient care and continuing professional development [9]. Even for those clinical hospital personnel who are concerned about the quality of the data, the most popular electronic source for information seeking was Google [10], as Google may be a portal to the MEDLINE or Pubmed database [10], one of the most popular and commonly used evidence-based medicine (EBM) electronic databases [11]. Thus, the Google search data can be expected to include searches by both health care professionals and nonprofessionals, making the user base extremely heterogeneous and sensitive to, for example, media trends.

We aimed to assess whether health care professionals’ use of Internet-based EBM databases was comparable to the use of the register of public primary health care diagnoses (Avohilmo) as a part of routine public health surveillance. Avohilmo serves as an electronic database for actual primary health care notifications collected from public sector health care units. Avohilmo data are used for example in health care decision making, planning, and research. When a patient visits the public primary health care unit in Finland, a physician makes a note of the diagnosis in the electronic patient record. From there the diagnosis will be transferred to the Avohilmo database maintained by the research and development institute, the National Institute for Health and Welfare (NIHW).

The Finnish Medical Society Duodecim is a scientific society [12] that publishes medical information and contributes to the continuous professional development of doctors in Finland. Duodecim Medical Publications Ltd, owned by the Finnish Medical Society Duodecim, carries out publication of medical information. Duodecim Medical Publications Ltd produces and maintains Internet-based, chargeable Physician’s databases (PD) consisting of, for example, practically orientated point-of-care medical guidelines especially for physicians serving in outpatient treatment and hospital outpatient clinics. The database is available throughout the entire health care system of Finland (over 20,000 working age physicians in Finland in 2014 [13]) and the users of database are health care professionals working in Finland. Every keyword that health care professionals search is included in a log file.

We chose Lyme borreliosis (LB) as a model to evaluate the usability of health care professionals’ queries for surveillance purposes in a dedicated EBM portal. LB is a spirochetal infectious disease caused by *Borrelia burgdorferi sensu lato* and is transmitted via ticks [14]. Seasonal variation is an important feature of LB [15,16], since annual climate changes affect tick activity that transmit the *B. burgdorferi* pathogen to humans between spring and autumn [16] when people go outdoors or on holiday. The incidence of LB has increased significantly in Europe [17,18]. Significant variation exists in both the temporal and the geographical distribution of LB in Finland [19] (personal communication by E Sajanti, MJ Virtanen, J Hytönen, J Sane, October 10, 2016). The differential diagnostics of LB presenting with erythema migrans (EM) are scarce [14], making register-based surveillance possible. The Avohilmo data on LB (mainly EM, ie, a typical tick-bite rash in LB) are registered by an International Classification of Diseases (10th Revision; ICD-10) disease classification code “A69.2” and are available since 2011. According to Avohilmo and NIDR registers, the incidence of LB in Finland has increased steadily (personal communication by E Sajanti, MJ Virtanen, J Hytönen, J Sane, October 10, 2016). Of note, in primary health care patients with EM, a clinical picture is sufficient for diagnosis and further laboratory testing is not recommended. Therefore, the cases reported to Avohilmo are not likely reported in NIDR. Our hypothesis was that the timeliness of health care professionals’ queries coincides with Avohilmo findings of LB. The chosen registers, PD and Avohilmo, represent unique databases, which do not allow matching the searches and diagnoses to one another.

## Methods

We carried out a descriptive register study to compare two registers, PD and the register of primary health care outpatient diagnoses (Avohilmo), to research health care professionals’ Internet-based queries of LB and public primary health care outpatient diagnoses on LB. To study the use of electronic databases in the context of a well-defined disease, we retrospectively collected logs of PD searches for LB by using the keywords: “borre*” or “lyme*” or “migrans*.” These searches were further defined by the number of searches (years 2011-2015) in the whole country and all 21 health care districts in Finland annually and monthly. Three high-incidence LB regions (Helsinki and Uusimaa, Southwest Finland, and Kymenlaakso), all located in Southern Finland [19], were selected to be studied further. We chose blood pressure and diabetes to serve as comparison words to LB search word to distinguish actual increase in single search parameter from annual increase of general searches. The aim of this study was to compare PD searches to public primary health care outpatient diagnoses (Avohilmo) (1) annually in the whole country during 2011-2015, (2) monthly in the whole country during 2014-2015, and also (3) annually in three selected high-incidence LB regions during 2011-2015. An ethical approval for this study was granted by NIHW.

## Results

**Figure 1:**
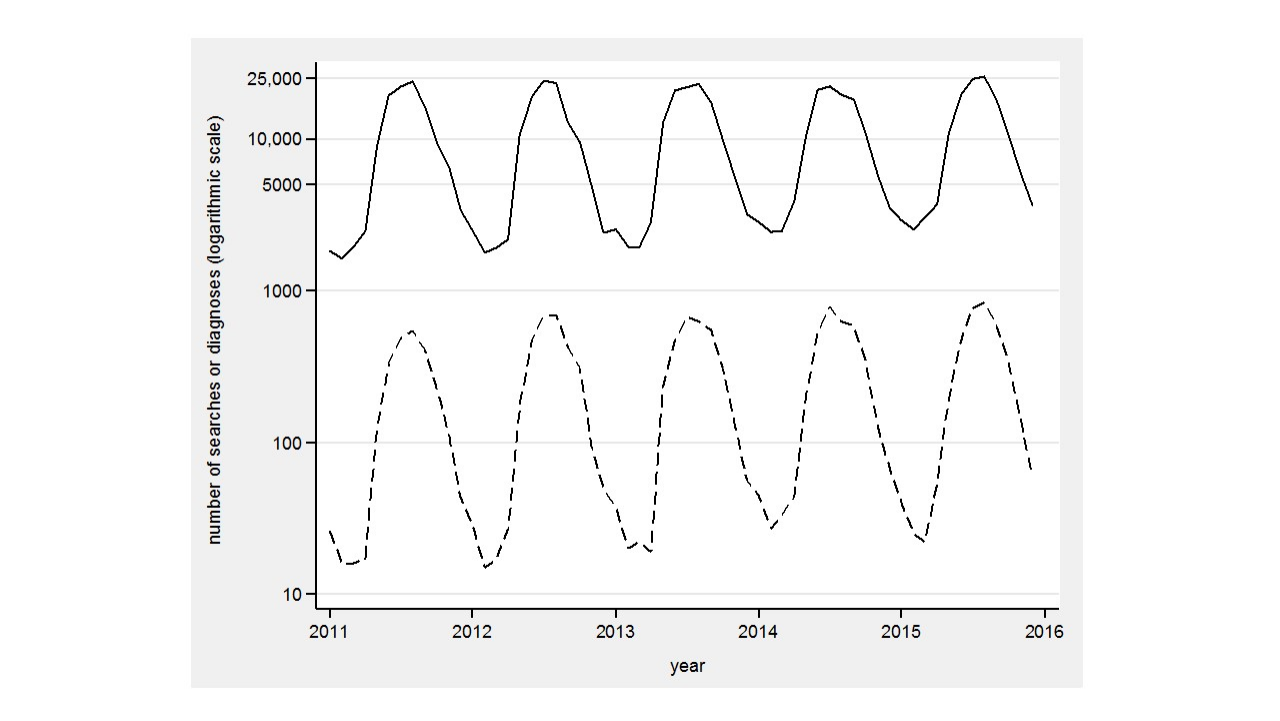
Physician's database searches for Lyme borreliosis (solid line) and Avohilmo diagnoses for Lyme borreliosis (dashed line) in the whole country during 2011-2015.

We found visually similar patterns in annual and regional variation in LB searches and primary health care outpatient diagnoses. Figure 1 shows the annual variation of the whole country in 2011-2015. The PD searches of the whole country in 2011-2015 start mostly in April and reach the maximum (searches peak at 25,463 and primary health care outpatient diagnoses at 835 in 2015) in July-August, and descend to the minimum in February (searches lowest at 1618 in 2011 and primary health care outpatient diagnoses at 15 in 2012). In June-September 2014, a plateau stage occurs, a pattern that is also present in Avohilmo. Comparison words, blood pressure and diabetes, to LB search word show no temporal variation (data not shown) as LB does.

**Figure 2:**
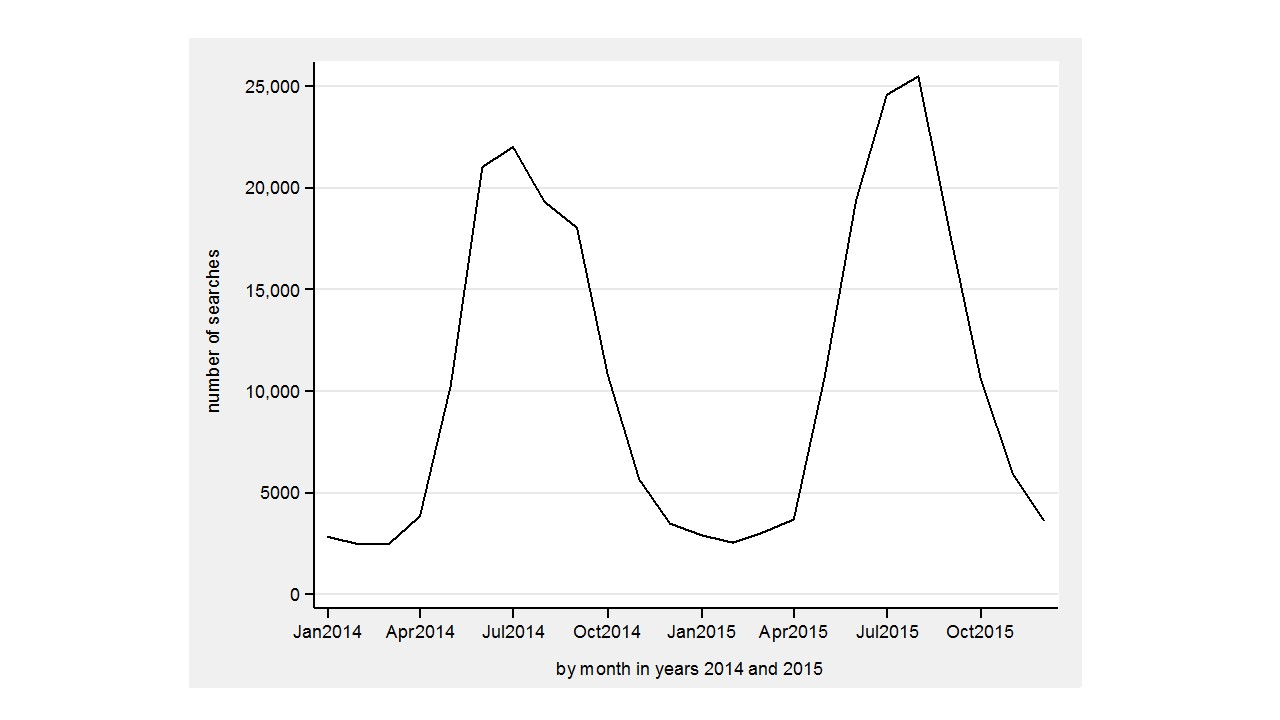
Physician's database searches for Lyme borreliosis in the whole country during 2014-2015.

**Figure 3:**
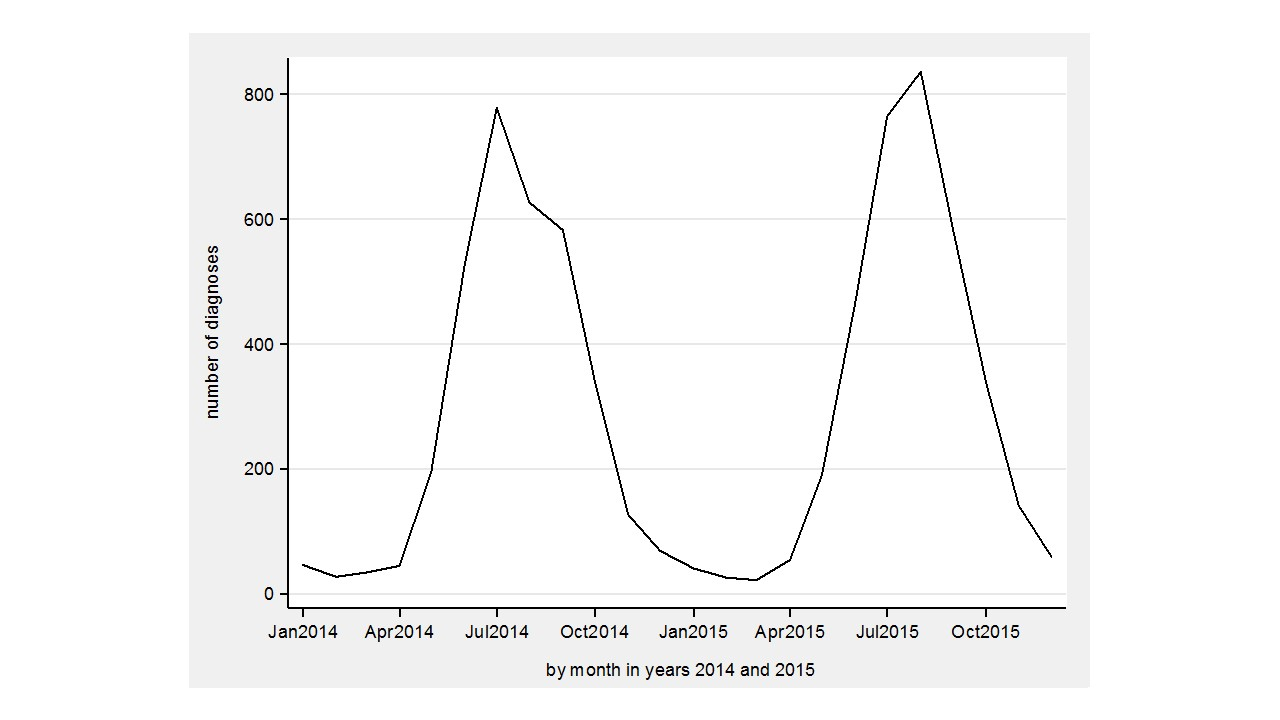
Avohilmo diagnoses for Lyme borreliosis in the whole country during 2014-2015.

The monthly variation in searches in the whole country in 2014-2015 is seen in Figure 2 and diagnoses in Figure 3. In 2014, the PD and Avohilmo data show a rapid ascent in April, both peak in July, and then start a decline forming a double-peaked pattern in July-September. The findings in PD and Avohilmo data reach the minimum in February and March 2015, respectively. In 2015, both searches and primary health care outpatient diagnoses show a rapid up-and-down pattern starting in April, peaking in August, and then declining fast.

In both PD and Avohilmo data, three hospital regions stand out as high-incidence areas of LB (Helsinki and Uusimaa, Southwest Finland, and Kymenlaakso). These regions show similar patterns between in both PD and Avohilmo data during 2011-2015 (data not shown). These regions also follow the same annual variation pattern as shown in Figure 1.

## Discussion

Our study showed visually similar patterns between PD searches and primary health care outpatient LB diagnoses both in the whole country and in three healthcare districts with high incidence of LB during 2011-2015. In PD searches, the seasonal variation was clear and regional differences appeared which were consistent with LB diagnoses registered in Avohilmo. A double-peaked pattern in July-September 2014 (Figures 2 and 3) may possibly be related to media trends.

The use of Internet-based EBM sources, such as the Cochrane Library, Cumulative Index to Nursing and Allied Health Literature (CINAHL), and MEDLINE or Pubmed, has increased over the years among health care professionals [20]. Log files from Internet portals such as Google could be utilized to help in detecting and predicting epidemiological patterns [1,4,5]. Although we speculate that the double-peaked pattern, formed by health care professionals’ searches, is related to media trends, we hypothesize that nonprofessionals’ queries are even more affected. Therefore, we believe professional queries to mimic more closely actual disease trends. The Google flu-related search trends that included searches by health care professionals and nonprofessionals appeared to fail to predict the timing of emerging infectious diseases [4]. Search logs from Internet EBM databases for health care professionals, however, are here shown to accurately mirror LB diagnoses in a nationwide discharge register.

Our study includes some limitations that should be considered. LB is often diagnosed days after the initial tick bite. It is not uncommon for a patient to seek physician’s attention after coming home from a holiday in a region with a high prevalence of LB. Therefore, the weakness in our study is associated with the fact that diagnoses and searches are not necessarily stated and performed at the same geographical location of contraction of disease. Another limitation in our study is that PD searches and Avohilmo diagnoses represent unique entries in these registers, not unique users or patients. Therefore, they cannot be linked directly to one another. Thus, the conclusions on the number of diagnoses should be carefully drawn when considering a measure of LB incidence. Our study showed strengths in representativeness (health care professionals) and timeliness (real-time Internet database).

To our knowledge, this is the first study to demonstrate an association between health care professionals’ Internet-based searches and a nationwide primary care discharge register on a specific diagnosis. We state that PD searches closely mimic the known seasonal variation of LB. This finding could be used for means to strengthen the surveillance of seasonal increases of the disease. Future research should focus on the validation of the method and the applicability of the method to other specific pathogens.

## Abbreviations

Avohilmo: the register of public primary health care diagnoses
EBM: evidence-based medicine
EM: erythema migrans
LB: Lyme borreliosis
NIDR: National Infectious Diseases Register
NIHW: National Institute for Health and Welfare
PD: Physician’s databases
